# Retinoic acid elicits a coordinated expression of gut homing markers on T lymphocytes of Zambian men receiving oral Vivotif, but not Rotarix, Dukoral or OPVERO vaccines

**DOI:** 10.1016/j.vaccine.2018.04.083

**Published:** 2018-06-27

**Authors:** Mpala Mwanza-Lisulo, Mumba S. Chomba, Mubanga Chama, Ellen C. Besa, Evelyn Funjika, Kanekwa Zyambo, Rose Banda, Mercy Imikendu, Sandie Sianongo, Robert E.W. Hancock, Amy Lee, Roma Chilengi, Andy J. Stagg, Boniface Namangala, Paul M. Kelly

**Affiliations:** a Tropical Gastroenterology & Nutrition Group, Department of Medicine, School of Medicine, University of Zambia, Lusaka, Zambia; b Department of Chemistry, University of Zambia, Lusaka, Zambia; c University of British Columbia, Vancouver, Canada; d Centre for Infectious Disease Research in Zambia (CIDRZ), Lusaka, Zambia; e Blizard Institute, Barts & The London School of Medicine, Queen Mary University of London, London, UK; f Institute of Distance Education, University of Zambia, Lusaka, Zambia

**Keywords:** ATRA, all-trans retinoic acid, RA, retinoic acid, CCR9, chemokine receptor 9, WGLF, whole gut lavage fluid, Retinoic acid, α4β7, CCR9, Gut mucosa, Vaccines

## Abstract

All-trans retinoic acid (ATRA) up-regulates, in laboratory animals, the expression of the gut homing markers α4β7 integrin and CCR9 on lymphocytes, increasing their gut tropism. Here, we show that, in healthy adult volunteers, ATRA induced an increase of these gut homing markers on T cells *in vivo* in a time dependent manner. The coordinated increase of α4β7 and CCR9 by ATRA was seen in 57% (12/21) of volunteers and only when given together with an oral Vivotif vaccine. When this coordinated response to ATRA and Vivotif vaccine was present, it was strongly correlated with the gut immunoglobulin A (IgA) specific response to vaccine LPS (ρ = 0.82; P = 0.02). Using RNA-Seq analysis of whole blood transcription, patients receiving ATRA and Vivotif in conjunction showed transcriptomic changes in immune-related pathways, particularly including interferon α/β signaling pathway, membrane-ECM interactions and immune hubs. These results suggest that exogenous ATRA can be used to manipulate responses to a subclass of oral vaccines, so far limited to a live attenuated Vivotif vaccine.

## Highlights

• ATRA increased vaccine specific IgA in gut secretions to Vivotif but not Dukoral or Rotarix.
• ATRA increased α4β7 and CCR9 gut marker expression in a coordinated manner only when given simultaneously with Vivotif vaccine.
• In individuals with coordinated gut marker expression Vivotif specific IgA increase was much stronger.

## 1 Introduction

There is strong evidence of diminished immunogenicity and efficacy of oral vaccines in developing country populations [1]. For example, oral rotavirus vaccine affords excellent (80–90%) protection against severe rotavirus diarrhea in industrialized and middle income countries but has been less efficacious when tested in infants in the developing world [2], [3]. The trivalent oral polio vaccine (OPVERO) has also been found to be less effective in children in the developing world [4] with estimated efficacies of around 21% in India compared to 50% in the United States [5]. In view of this, there is an urgent need to identify ways to improve immune responses of these oral vaccines in the settings where they are most needed.

Vitamin A supplementation significantly reduces mortality from diarrheal infections as evidenced by results of several studies [6], [7], [8], [9]. The mechanism of protection however is unclear [6], [9].

Vitamin A is oxidized to four isoforms of retinoic acid (RA); these include all-trans RA (ATRA), 9-cis RA, 13-cis RA, and 11-cis RA. The RAs have important immunological functions especially on intestinal defense and mucosal immune responses.

Selective migration of the effector T cells to the gut requires the expression of α4β7-integrin and the chemokine receptor, CCR9. Integrin α4β7 is the homing receptor for the small and large intestine, whereas CCR9 is the small intestine-specific homing receptor [10]. Several studies have shown that RA is a key mediator of T cell homing to the gut [11], [12], [13]. Iwata and colleagues [11] demonstrated that CD4^+^ T cells activated in vitro in the presence of ATRA had enhanced expression of the gut-homing receptors α4β7-integrin and CCR9. Hammerschmidt and colleagues [12] demonstrated that RA-assisted subcutaneous immunization resulted in pronounced localization of antigen-specific plasma cells in the small intestine and a robust immunoglobulin A (IgA) response in serum and intestinal washes. In recent preliminary work [14], we found that ATRA could enhance gut IgA responses to Vivotif typhoid vaccine in intestinal washes. Collectively these observations suggest that RA-assisted immunization generates intestinal immunity and confers the benefits of mucosal protection.

Retinoic acid has been known to affect expression of the polymeric immunoglobulin receptor (pIgR) [15] which transports polymeric immunoglobulin A (pIgA) into external secretions as secretory IgA (S-IgA). This suggests that vitamin A might be required for the proper regulation of IgA transport in response to mucosal infection, which would be a desirable characteristic of an orally-active adjuvant [16], [17].

The present study investigated whether the ATRA adjuvanticity seen with Vivotif vaccine in African men could be generalized to other oral vaccines. We asked if ATRA given alongside oral vaccines could improve the immune response by enhancing expression of α4β7-integrin and CCR9 gut homing molecules, and if so, under what circumstances.

## 2 Methods

### 2.1 Recruitment

We recruited 94 adult male volunteers aged between 18 and 60 years from a high- density township in Lusaka, Zambia, as previously described [14]. A 3-stage consent process was used [20], [21]. Nutritional assessment and HIV testing was done. Details in Appendix A, [27], [28], [29], [30], [47], [48], [49], [50] methods.

### 2.2 Ethical Approval

Approval for the study was obtained from the Biomedical Research Ethics Committee of the University of Zambia (references 012-06-12 and 013-01-14).

### 2.3 Vaccine administration and sample collection

Recruited volunteers (n = 94) were randomized to receive one of four oral vaccines: Vivotif (Crucell, Switzerland) [22], [23], Dukoral (SBLVaccines), Rotarix (GSK), and OPVERO (Sanofi Pasteur). Each vaccine was given with or without ATRA on day 1. ATRA was administered as 10 mg daily for 8 days and 1 h before vaccination. Vivotif was given on days 1, 3 and 5 while Dukoral, Rotarix and OPVERO were administered as single doses. Whole gut lavage fluid was collected as previously described [14], [25] on days 0 and 14 (see supplementary material methods). Blood was also collected at the same time-points for the serum antibody assays.

### 2.4 Detection of Vivotif and Dukoral-specific IgA and IgG in serum and gut lavages

Vivotif and Dukoral LPS antigen preparations were made using a modified phenol extraction technique as previously described [14], [22]. Analysis of vaccine specific antibodies in serum and gut lavages was performed as described previously [14].

### 2.5 Detection of Rotarix-specific IgA and IgG in serum and gut lavages

Analysis of IgA and IgG antibody responses was done using ELISA (see supplementary material).

### 2.6 Detection of OPVERO-specific IgA and IgG in serum

Analysis of OPVERO-specific antibodies in serum was performed by neutralization.

### 2.7 Analysis of effects of ATRA on polymeric Ig receptor (pIgR)

Biopsies (<20 mg of tissue) were collected from the jejunum of 13 participants and analyzed for the effect of ATRA on expression of pIgR (for details refer to Supplementary material methods).

### 2.8 Analysis of baseline serum retinol concentrations

Retinol in serum was assayed by high performance liquid chromatography (HPLC) modified version [26].

### 2.9 Transcriptome analysis of effects of ATRA during vaccination

Blood samples were collected from 8 male individuals given both ATRA and Vivotif on day 0 (baseline) and day 8. Each sample comprised 2.5 ml of blood directly drawn into a PAXgene blood RNA tube (Qiagen), inverted 10 times, then kept at room temperature for 2 h before being stored at −80 °C until RNA extraction (see Supplementary material methods for more details).

### 2.10 Statistical analyses

All graphical and statistical tests were performed using GraphPad Prism 6.0 (La Jolla, CA) and STATA 12 (Stata Corp, College Station, TX, USA). Statistical comparisons were done using nonparametric tests (Mann-Whitney, Kruskal-Wallis and Spearman’s rank correlation coefficient). Gut lavage responses are expressed as change in log titer values; P values <0.05 were assumed significant.

## 3 Results

In total, 94 participants were randomized to receive one of 4 vaccines and participant demographics are described in Table 1. The HIV prevalence in this study cohort was 21% with average age being 29 years and nutritional status comparable in each group. We report combined data for HIV infected and non-infected, as we found HIV infection had no effect on the intestinal IgA response to Vivotif (Supplementary Fig. 1).

**Table 1:** Baseline characteristics of study participants.

Number	33	13	7	6	4	5	7	6	13	P
Vaccine	Vivotif	Vivotif	Rotarix	Rotarix	Opvero	Opvero	Dukoral	Dukoral	None	
ATRA	+	−	+	−	+	−	+	−	+	
Age (years)	34 (22–42)	38 (29–40)	21 (19–25)	39 (27–53)	29 (25–33)	20 (19–42)	31 (28–40)	39 (37–40)	28 (22–43)	0.43
HIV Positive (n)	5	2	0	1	1	1	3	4	3	
BMI (kg/m^2^)	20 (19–23)	22 (19–23)	20 (19–21)	20 (19–20)	21 (19–24)	19 (17–25)	20 (18–24)	19 (18–19)	19 (18–24)	0.43

The number of participants randomised to Vivotif includes those in the time course experiments, vaccine comparison experiments and pIgR experiments. Volunteers were all healthy adult men randomised to receive one or none of the vaccines with or without 10 mg ATRA given daily for 8 days. For vaccine comparisons 8 volunteers were recruited, pIgR studies had 13 volunteers while the time course studies had 12 volunteers all given Vivotif + ATRA. These are shown as pooled Vivotif vaccine groups. Continuous variables are shown as median and interquartile range (IQR). ATRA: all-trans retinoic acid, BMI: body mass index.

### 3.1 ATRA increased gut IgA directed at Vivotif LPS, but not other vaccine antigens

**Fig. 1:**
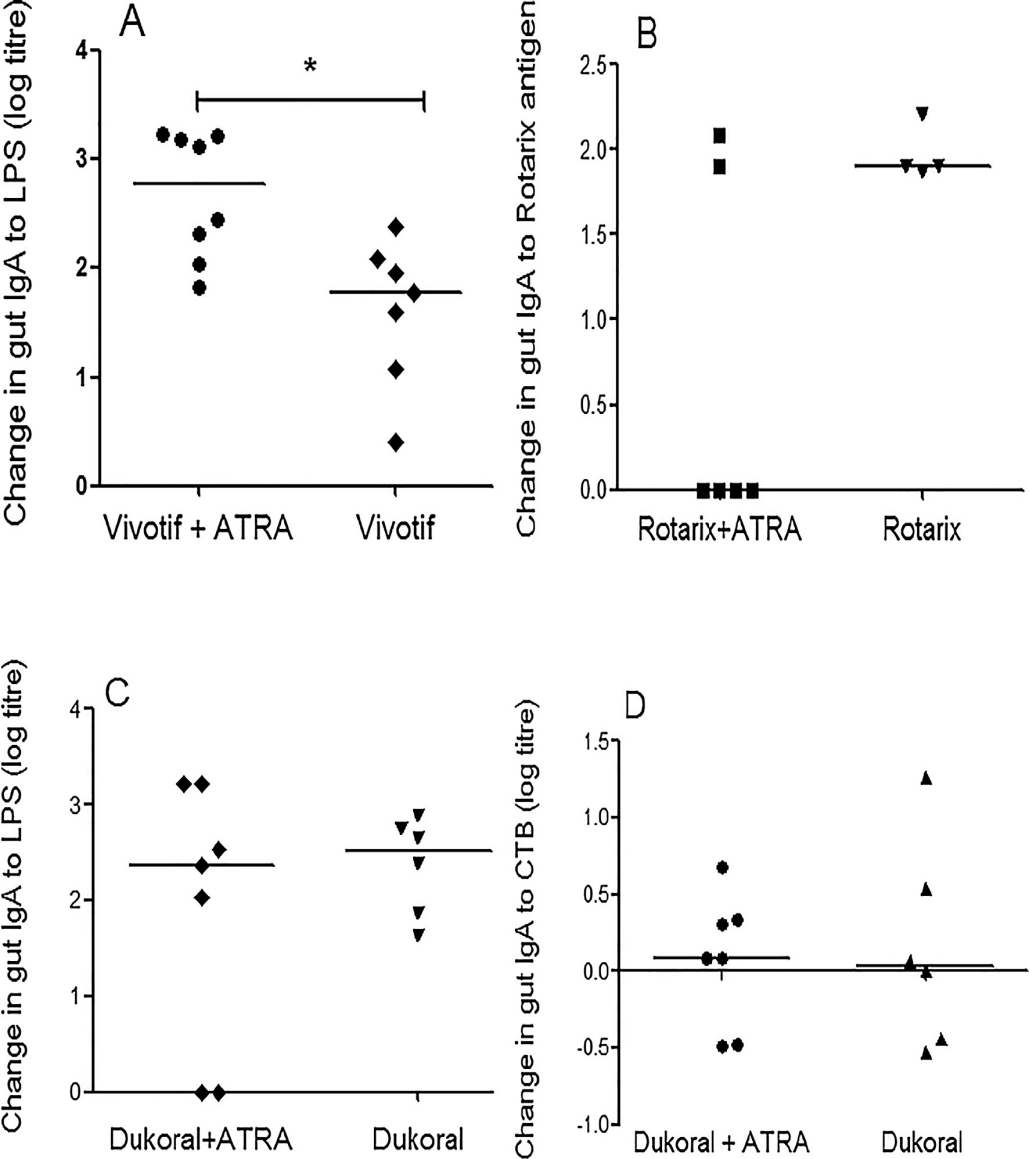
Change in specific IgA in intestinal lavage fluid in volunteers given one of three vaccines. ‘Change’ is the difference in log titre values of IgA pre- and 14 days post vaccination. Antigen-specific IgA responses to Vivotif and Dukoral LPS, Dukoral CTB and Rotarix antigen were measured in whole gut lavage fluid (WGLF). (A) Specific IgA in WGLF against Vivotif LPS was increased (^*^P = 0.01) in Vivotif recipients who received ATRA. No change was seen in responses to the other vaccine antigens: (B) Rotarix (P = 0.19), (C) Dukoral LPS (P = 0.83) or (D) Dukoral Cholera Toxin B subunit (CTB) (P = 0.84). Data were analyzed using Mann-Whitney test. Responses are shown measured as change in log titers.

Antibody titers to Vivotif, Dukoral and Rotarix were measured as changes in log titers in response to vaccination with or without daily administration of 10 mg ATRA for 8 days. Antibody titers in whole gut fluid against Vivotif LPS antigen were significantly increased (Fig. 1A) in the group that received Vivotif vaccine as well as ATRA, consistent with our previous findings [14]. There was no significant change in mucosal responses to Rotarix antigens or Dukoral (whether LPS or CTB) antigens in gut lavage samples (Fig. 1B-D). Antibody titers in serum showed no significant change in any of the vaccine groups (Supplementary Fig. 2). This increase in specific IgA was not a consequence of increased pIgR expression in intestinal biopsies (Supplementary Fig. 3).

### 3.2 Effect of ATRA was vaccine and time dependent

**Fig. 2:**
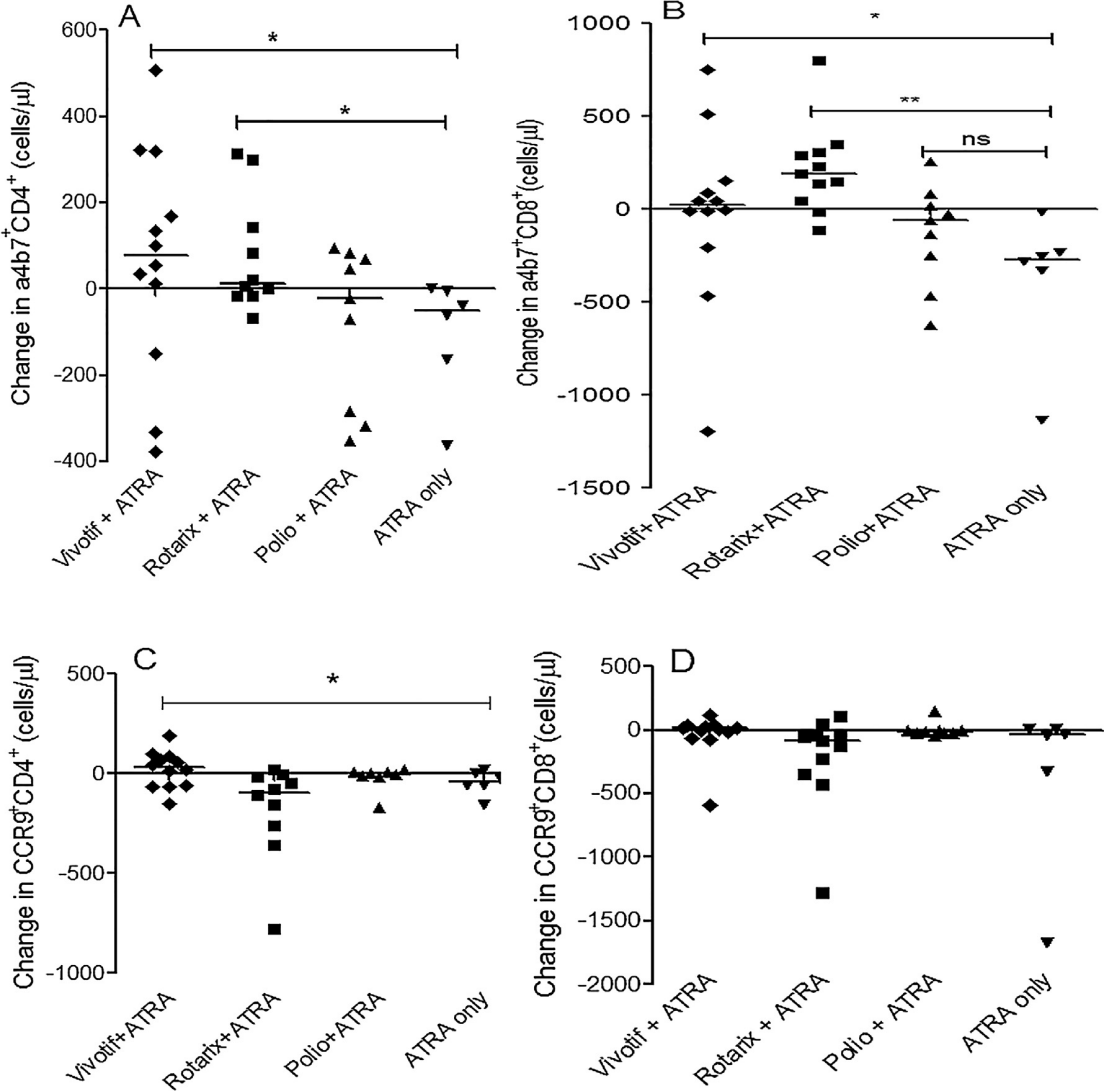
Summary of changes on CD4^+^ and CD8^+^T cells expressing α4β7 and CCR9. Volunteers received either one of 3 vaccines (Vivotif, Rotarix or Opvero) plus ATRA or ATRA alone. (A) Change in α4β7 expression on CD4^+^ T cells was significantly higher in participants that received Vivotif with ATRA (^*^P < 0.05) or Rotarix with ATRA (^*^P = 0.02). (B) Change in α4β7 expression on CD8^+^ T cells was also significantly higher in the group that received Vivotif with ATRA (^*^P = 0.03) or Rotarix with ATRA (^**^P = 0.002). (C) Change in CCR9 expression on CD4^+^ T cells was significantly higher in participants that received Vivotif with ATRA (^*^P = 0.02). (D) CCR9 expression on the CD8^+^ T cells; no effect of ATRA was observed. The changes were calculated by subtracting the pre- vaccination CD4^+^ T cells expressing the gut homing markers of interest from the post vaccination CD4^+^ T cells. The effects of ATRA were analyzed using the Kruskal-Wallis test.

We then analyzed whether expression of gut homing markers was vaccine dependent. We found that there was an increase in α4β7^+^CD4^+^ (Fig. 2A) and α4β7^+^CD8^+^T cells (Fig. 2B) in the Vivotif and Rotarix groups when compared to those that received only ATRA. We only observed slightly enhanced CCR9^+^ CD4^+^T cells (Fig. 2C) but not CCR9^+^CD8^+^T cells (Fig. 2D) in those given both Vivotif and ATRA. When analyzing only activated (HLA-DR+) CD3+ T cells, it was the CD8+ cells which showed the greatest change in α4β7 and CCR9 expression (Supplementary Fig. 4). Conversely, a slight decrease in α4β7^+^ DR^+^CD4 ^+^T cells was observed at day 3 (*P* = 0.02) and day 8 (*P* = 0.01) in participants that received both Vivotif and ATRA (Supplementary Fig. 5). These data indicate that the effect of ATRA is seen only in the presence of antigen and on day 14 post vaccination.

### 3.3 ATRA caused a coordinated response of α4β7 integrin and CCR9 only if given simultaneously with Vivotif vaccination

**Fig. 3:**
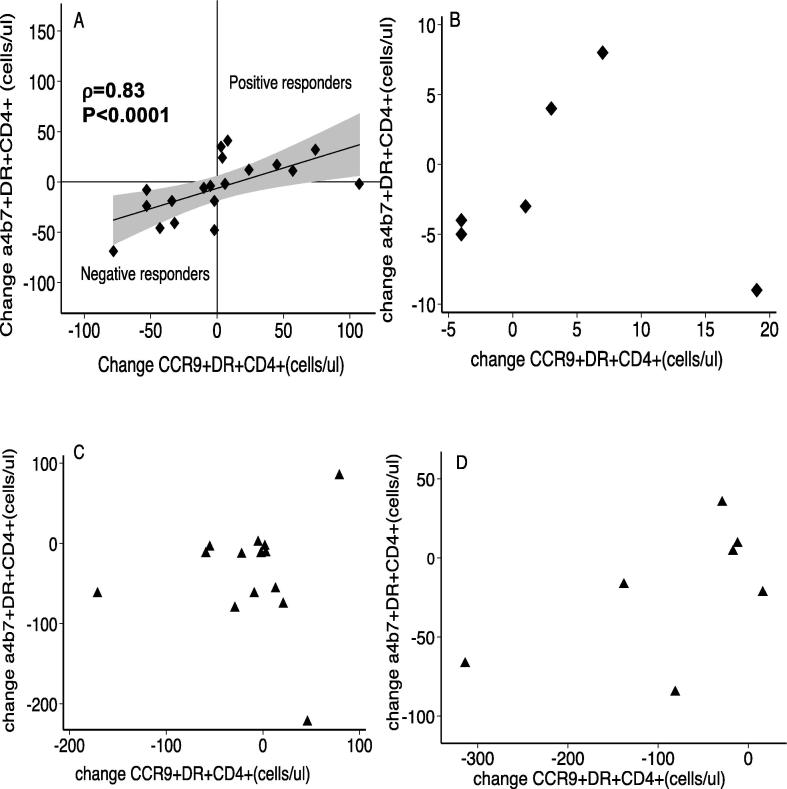
ATRA induced coordinated expression of α4β7-integrin and CCR9 on CD4+ T cells in Vivotif recipients. (A) Change in α4β7+CD4+ and CCR9+CD4+ was closely correlated (ρ = 0.83; P < 0.0001) in participants that received Vivotif with ATRA. Participants given (B) Vivotif alone (ρ = 0.12; P = 0.83), (C) ATRA alone (ρ = 0.07; P = 0.82), or (D) Rotarix with ATRA (ρ = 0.39; P = 0.38) showed no correlation in gut marker expression respectively. Correlations were analyzed using the Spearman rank correlation test.

Having established that the co-administration of Vivotif and ATRA resulted in increased α4β7^+^CD4^+^ and CCR9^+^CD4^+^ T cells at day 14, we explored the correlation of these two gut-homing markers. Spearman’s rank test revealed a strong correlation (ρ = 0.83; P < 0.0001) of these gut homing markers on CD4^+^T cells (Fig. 3A), but only when ATRA was given alongside Vivotif. We designated those individuals with a coordinated increase in these two gut homing markers as *positive responders* and those with a coordinated decrease as *negative responders*. A coordinated increase in both gut homing markers was observed in 57% (12/21) of volunteers that received Vivotif and ATRA simultaneously. This effect was not seen with Vivotif alone (Fig. 3B), ATRA alone (Fig. 3C) or Rotarix given with ATRA (Fig. 3D). Our data therefore suggest that the coordinated gut homing response to ATRA is restricted to the situation when co-administered with Vivotif.

### 3.4 Baseline serum retinol was lower in individuals that showed coordinated responses to α4β7 integrin and CCR9

Baseline serum retinol concentrations (Table 2) in the *positive responders* were significantly lower (median of 1.41 µmol/L, interquartile range 1.06–2.48 µmol/L) than the retinol concentration in the *negative responders* (median 2.68 µmol/L, interquartile range 1.68–3.29; *P* = 0.03). In the *positive responder* group, 40% (4/10) of the subjects had serum retinol concentrations of ≤1.05 µmol/l, consistent with vitamin A deficiency, compared to 0% (0/7) of *negative responders*.

**Table 2:** Baseline characteristics of positive responders and negative responders.

Characteristic	Positive responders	Negative responders	P
n	12	9	
Sex	Male	Male	
Age (mean, years)	36	29	
HIV seropositive (n)	3	2	
Serum retinol (μmol/L)	1.4 (1.06–2.48)	2.68 (1.68–3.29)	0.03

A retinol concentration of 1 μmol/L was equivalent to 28.

### 3.5 The change in α4β7 on DR^+^ CD4^+^ T cells correlated strongly with the intestinal IgA response to Vivotif

**Fig. 4:**
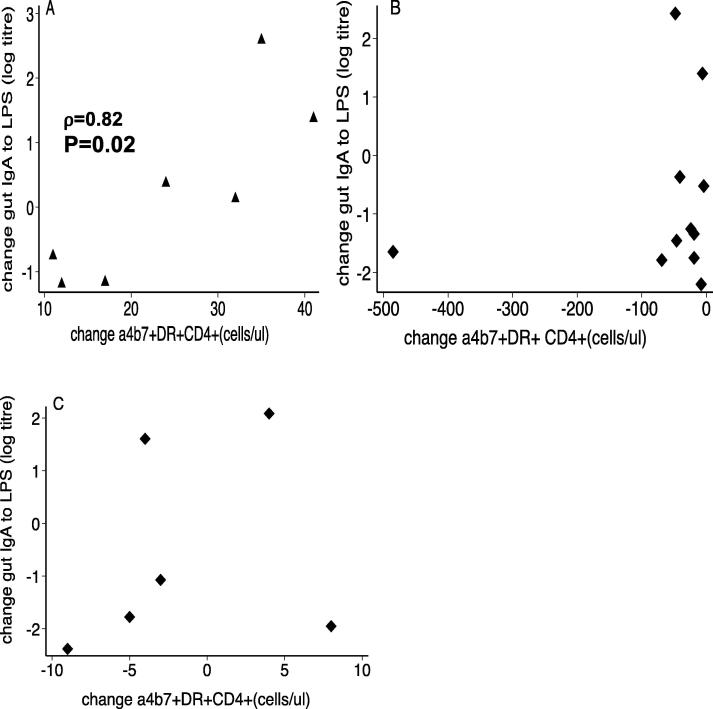
Correlation of change in gut IgA to change in α4β7^+^DR^+^CD4^+^ cells in the positive and negative responders. (A) The positive responders showed a strong correlation (ρ = 0.82; P = 0.02) of gut IgA to gut marker expression. (B) This was not observed in the negative responders (ρ = 0.31, P = 0.2) or those given (C) Vivotif alone (ρ = 0.37; P = 0.2). Correlations were analyzed using the Spearman rank correlation test.

We then looked for a correlation between the change in gut homing marker expression in blood and the intestinal IgA response to Vivotif (i.e. gut lavage IgA to Vivotif LPS). The change in α4β7 marker expression on CD4^+^T cells was strongly correlated with the change in specific IgA response to Vivotif LPS in the intestine in the *positive responders* only (Fig. 4A). In *negative responders* (Fig. 4B), or those given Vivotif alone (Fig. 4C), there was no correlation.

### 3.6 Transcriptome analysis

To elucidate the transcriptional changes that occurred during co-administration of ATRA and the oral vaccine Vivotif, we performed RNA-Seq on blood samples. Transcriptomic changes in immune-related pathways, particularly including interferon α/β signaling pathway, membrane-ECM interactions and immune hubs were observed (Supplementary Table 1). Key immune hubs identified include fibronectin 1 (FN1), AXL Tyrosine-protein kinase receptor, complement component 1 (C1QB), complement component 4 binding protein (C4BPA) and HLA-DRB1 (Supplementary Fig. 6).

## 4 Discussion

The global burden of diarrheal disease and the reduced efficacy of oral vaccines in populations from developing nations have highlighted the need for new strategies for vaccination against diarrheal diseases, for example by using oral adjuvants. This study has revealed four important effects of ATRA on immune responses to oral vaccination: First, we have confirmed earlier findings [14] that ATRA can increase gut mucosal IgA directed at an oral live attenuated typhoid vaccine, and that this increase is not attributable to the increased pIgR expression as had been suggested previously [15]. Secondly, our data suggests that ATRA can increase the number of circulating CD4^+^ T cells expressing the gut homing markers α4β7 and CCR9 in a coordinated and vaccine-specific fashion in a subset of individuals. Thirdly, integrin α4β7 increase was strongly correlated with the intestinal IgA responses in *positive responders,* i.e. those individuals with a coordinated increase in the gut homing markers. Lastly, we found that ATRA treatment may cause changes in immune related pathways, including interferon α/β signaling pathway (adjusted p-value = 0.0015), membrane-ECM interactions, and immune hubs including fibronectin 1, AXL kinase, complement pathway components and major histocompatibility complex components such as HLA-DRB1. Since these elements were impacted by the end of the 8 day ATRA treatment, we propose that they are responsible, in part, for the increased responses to Vivotif vaccine.

We have demonstrated previously [14] that, in whole gut fluid, ATRA is able to enhance specific IgA against vaccine antigens suggesting that ATRA could play an adjuvant role when given alongside Vivotif. To determine whether the adjuvanticity of ATRA could be translated to other oral vaccines, we evaluated its effect on three other vaccines; Rotarix, Dukoral and OPVERO vaccines, all orally administered one hour after administration (or not) of 10 mg ATRA given daily for 8 days. Our data revealed that ATRA adjuvanticity in this study only occurred with Vivotif and not the other vaccines, suggesting that the ATRA effect might be vaccine and/or antigen specific and not a generalizable adjuvant property. There may be several contributors to the specificity of ATRA in promoting adjuvanticity with Vivotif and not the other oral vaccines. The most important factor is likely to be the type of immune responses being elicited by the vaccines. Vivotif induces a vigorous T-cell response that favours the production of interferon gamma (IFN-ɣ), tumour necrosis factor-α (TNF-α), interleukin -1 (IL-1) and IL-6, indicative of a Th1-type response probably through Class II antigen presentation [31], [32]. Rotavirus has been shown to drive the secretion of TGF-β resulting in inhibition of Th1 responses by dendritic cells [33]. In one study [34], Rotarix vaccination showed no discernible effect on IFN-ɣ and TNF-α response in a considerable proportion [76% (16/21)] of vaccinees. Another study [35] showed that oral polio vaccine was associated with down regulation of cytokine (IFN-ɣ and TNF-α) production when co-administered with BCG. The authors speculated that polio virus might have specific immune modulatory molecules that down-regulate immune responses to antigens to which immune responses are being mounted simultaneously. Finally, the inactivated bacterial vaccine Dukoral tends to elicit diminished or skewed CD4^+^ T cell responses towards development of a Th2 T cell phenotype because of the presence of CTB [36]. Of the vaccines studied, Vivotif was the only vaccine that is likely to elicit a Th1-type immune response through Class II antigen presentation. Intriguingly, ATRA led to an increase in several MHC class II components including a major component enabling antigen recognition by T cells, HLADRB1. We hypothesize here that it is the nature of the context of antigen presentation, in this case for a vaccine that generates an IFN-ɣ dominant response, which determines whether ATRA has an effect [37]. Also, in a pro-inflammatory context involving cytokines, such as IL-15, ATRA probably acts through dendritic cells to decrease conversion of naïve T cells into T regs and to enhance Th1 cell polarization. However, this hypothesis will have to be tested empirically by inclusion of another vaccine group that elicits a Th1 type response, and that the experimental protocol would need to include direct demonstration of Th1 and Tregs in comparison with Th2 and Th17 responses.

To our knowledge, this is only the second study to evaluate ATRA effects on immune function in humans *in vivo*, since most other studies [11], [13], [38], [39], [40], [41], [42], [43] have used in vitro systems or animal models. Our findings showed an increase in CD4 T cells expressing α4β7 and CCR9 following oral administration of 10 mg ATRA but only when given with Vivotif but not Rotarix or OPVERO vaccines. These results are consistent with other studies in animal models [11], [12], [39], [40].

There is also evidence that ATRA has effects on other cell types that also possibly migrate to the gut. Pantazi and colleagues [13] demonstrated in a murine model, that retinoic acid signaling in B cells is not essential for their homing to Peyer’s Patches but is necessary in generating antigen-specific IgA responses in the gut. ATRA has also been found to enhance the migratory properties of dendritic cells, which is crucial for their antigen presenting function during infection. We must emphasize that while it would have been of interest to extend the scope of our work to include B cells, NK cells and DCs, in a human study one is always constrained by the experimental procedures which we can conduct, acceptable volumes of blood to be drawn, and the invasiveness of endoscopic procedures. Our attention in this study was drawn to T cells because of the mouse work by other groups [11], [41], [42] and our own previous study [14] suggesting that ATRA can augment responses to Vivotif vaccine.

For safety reasons, one limitation of this study is that it was carried out only in men due to the teratogenicity of ATRA [18], [19]; and with no clear safe dose, ATRA could not be used in women who might become pregnant during the study. As other studies [44], [45] confirm differences between males and females in response to vaccination and we suspect that responses in women would have been different. A further limitation is that we did not analyse the gut-resident cells to see the effect of ATRA on the gut immune responses, as we did not have ethical approval to perform gut lavage and endoscopic biopsies on the same participants.

When the baseline serum retinol concentration was analyzed, we found that 4/10 (40%) of the *positive responders* had a clear or borderline vitamin A deficiency but none of the *negative responders* had such deficiency. The difference observed might signify that the effect of ATRA is seen predominantly in people with borderline vitamin A deficiency. Also, the data could suggest that the vitamin A deficiency seen in the positive responders may have resulted in the dendritic cells being altered toward being more inflammatory producing high levels of IL-13 and TNF-α [46] thus creating a favorable environment in which ATRA has an effect. The response to ATRA and Vivotif was also not explained by HIV infection. A high proportion of this variability therefore remains unexplained, as evident from our RNA-Seq studies, so the possibility of an immunogenetic predisposition needs to be considered in future work.

It must be pointed out here that ATRA is also obtained from vitamin A in the diet, and the authors did not control or attempt to control for diet in the cohort because of the recall bias that doing a food frequency questionnaire has. It is also apparent from pharmacological data [14] that oral administration of 10 mg ATRA over a period of 8 days generates a sharp rise in serum concentrations of ATRA required to produce the desired immune effects that were the focus of the current study. Also of note is that ATRA levels were not monitored longitudinally but only evaluated at two time points; baseline and day 14 post vaccination.

## Appendix A Supplementary material

Supplementary data 2

Supplementary Fig. 1 Changes in wglf IgA to Vivotif LPS in HIV seronegative and HIV seropositive vaccine recipients. No significant difference (P = 0.34) was seen in IgA responses between the two groups.

Supplementary Fig. 2 Change in specific IgA and IgG in serum of volunteers given one of 3 vaccines with or without ATRA. Antigen-specific IgA responses to Vivotif LPS, Dukoral LPS, Rotarix antigen and Opevero antigens were measured in serum. There was no significant difference in serum IgA in (A) Vivotif recipients who received vaccine with ATRA or no ATRA. No change was seen with the other vaccine antigens either (B) Dukoral LPS, (C) Rotarix antigen and (D) Opvero antigen. (E) Antigen-specific IgG responses in serum of Vivotif recipients showed no difference between the two groups. Opvero antigens: P1, P2 and P3.

Supplementary Fig. 3 Summary of changes in pIgR:CK19 ratio. Effect of ATRA on pIgR was measured at baseline and day 14 post vaccination. There was no significant difference in the change in pIgR:CK19 ratio post Vivotif vaccination alongside (or without) ATRA treatment.

Supplementary Fig. 4 Summary of changes on activated CD4^+^ and CD8^+^T cells expressing α4β7 and CCR9 in volunteers given one of 3 vaccines (Vivotif, Rotarix or Opvero) plus ATRA and those given ATRA alone.(A)Change in α4β7 expression on DR^+^CD8^+^ T cells was significantly higher in participants that received vaccine with ATRA: Vivotif with ATRA (^∗∗∗^P = 0.004), Rotarix with ATRA (^∗∗∗^P = 0.001) and polio with ATRA (^∗^P = 0.04).(B) Change in CCR9 expression on DR^+^CD8^+^ T cells was significantly higher only in the those that received Vivotif with ATRA (^∗^P = 0.03).There was no difference in (C) α4β7 or (D) CCR9 expression on DR^+^CD4^+^ T cells for all of the vaccine group.

Supplementary Fig. 5 10 mg ATRA given daily for 8 days following oral Vivotif vaccination enhanced total CD4^+^ T cell count, α4β7-integrin and CCR9 on gut homing lymphocytes. Participants given vaccine with ATRA showed a drop in α4β7 expression at day 3 (^∗^P = 0.021) and day 8 (^∗^P = 0.013) but increased at day 14 (^∗^P = 0.003). CCR9 expression (^∗∗∗^P = 0.0002) and total CD4 count (^∗∗^P < 0.0001) was also increased in the same group. Participants given vaccine only and ATRA only showed a drop in α4β7 expression (^∗^P = 0.025 and ^∗∗^P = 0.009 respectively) over 14 days. The total CD4 count in the vaccine only group also showed a significant drop (^∗∗^P = 0.001) at day 14. Asterisks (∗) indicate that the effect of ATRA over the indicated time course is statistically significant . Effects of ATRA over time were analsed using the 1-way ANOVA test.

Supplementary Fig. 6 Network analysis of differentially expressed genes using NetworkAnalyst. 56 differentially expressed genes identified in the blood of volunteers, by RNA-Seq, were analyzed by comparing day8 versus baseline (pre-immunization) of ATRA treatment alongside vaccination (identified by DESeq2 likelihood ratio test). Red genes are upregulated and green genes are downregulated and grey nodes are direct interactors of these genes. Key immune hubs identified include fibronectin 1 (FN1), AXL Tyrosine-protein kinase receptor, complement component 1(C1QB), complement component 4 binding protein (C4BPA) and HLA-DRB1 are highlighted with an orange ring.
