# Supplementary material for: Retinoic acid elicits a coordinated expression of gut homing markers on T lymphocytes of Zambian men receiving oral Vivotif, but not Rotarix, Dukoral or OPVERO vaccines
Source: Vaccine. 2018 Jun 27;36(28):4134–41. doi: 10.1016/j.vaccine.2018.04.083 (PMC6020133; doi:10.1016/j.vaccine.2018.04.083)
Supplement: Supplementary data 1 [file mmc1.doc]

**SUPPLEMENTARY MATERIAL METHODS**

**Recruitment.** We recruited 94 adult male volunteers aged between 18-60 years from a high- density township in Lusaka, Zambia, as previously described [14]. All participants were screened for helminth infection through a single stool sample examination using the Kato-Katz technique. Nutritional assessment of each volunteer was carried out by measuring the body mass index (BMI) and mid upper arm circumference (MUAC). HIV testing was also offered to the participants and required for inclusion. Participants were excluded if they gave a history of diarrhea within 1 month prior to study, had received any antibiotics within 2 weeks or had been vaccinated with any of the vaccines to be studied within 5 years of the study. All females were excluded from the study in view of potential teratogenicity and the risk to pregnant women [18,19].

**Ethical Approval**. Approval for the study was obtained from the Biomedical Research Ethics Committee of the University of Zambia (references 012-06-12 and 013-01-14). A 3-stage consent process was done: (1) house-to-house sensitization drive, (2) focus group discussions/ community discussions, where the purpose of the study was explained (3) face-to-face interviews; purpose of the study was explained in more detail followed by consent and recruitment [20,21]. Participation in the study was voluntary and the participants were invited to the laboratory facilities and were shown how their samples would be processed [21]. No financial incentives or other gifts were offered to participants except for transport refunds.

**Collection of WGLF.** Whole gut lavage fluid was used to assess mucosal responses as previously described [14,25] on days 0 (the day before vaccination) and 14. Following an overnight fast, gut lavage solution (Klean-prep; Norgine, Uxbridge, UK) was prepared as per manufacturer’s instructions and each participant drank on average 3 liters of gut lavage solution before passing watery stool that eventually cleared into a gut lavage effluent having minimal particulates. Approximately 50ml of effluent was collected into a 50ml centrifuge tube and 1 tablet of complete EDTA-free Protease inhibitor cocktail (Sigma) was added to each 50ml sample of gut fluid within 5-15minutes of collection to inhibit serine, cysteine and aspartic proteases.1ml of 0.5 M EDTA (Sigma) was also added immediately to a final concentration of 10mmol/l, inhibiting metalloproteases. Samples were spun in a refrigerated (4^o^ C) centrifuge at 4226 x g for 20 minutes and supernatant was aliquoted and stored at -80^o^C.

**Detection of Rotarix-specific IgA and IgG in serum and gut lavages.**

Nintey-six well microtiter plates (Thermo Scientific, Denmark) were coated with rabbit hyperimmune serum to rhesus rotavirus (RRV) at a 1: 10,000 dilution and incubated at 4^o^C overnight. The next day, plates were blocked with blocking buffer (phosphate buffer saline [PBS] supplemented with 5% skim milk), incubated at 37^o^C, and were subsequently treated with a 1:10 dilution of supernatant from clarified Rotarix vaccine. Gut lavage and serum samples were diluted serially in diluent buffer (PBS supplemented with 1% skim milk and 0.5% [vol/vol] of 10% polyoxyethylene ether (W1)). Following a 2hour incubation, antibodies were detected with biotin-labeled affinity purified antibody to human IgA (dilution of 1:2000) or IgG (dilution of 1:3000) (Kirkegaard and Perry Laboratories, USA), followed by ExtraAvidin (dilution of 1:3000), then visualized by incubation with tetramethylbenzidine (TMB, Sigma). Plates were read using the MRXe plate reader at 450 nm.

**Analysis of effects of ATRA on polymeric Ig receptor (pIgR)**

Analysis of effects of ATRA on polymeric Ig receptor (pIgR) Briefly, the biopsy tissue from the jejunum was homogenized and centrifuged at 14000 rpm for 3 minutes. The supernatant was mixed with 700 µl of 70% ethanol and centrifuged at 8000 x g for 15 seconds. The flow through was discarded and sample washed with 700 µl of buffer RW1 once and twice with 500 µl of buffer RPE. RNA was eluted at 8000 x g for 1 minute with 35 µl of RNase-free water. An extraction control was run after every fourth sample. The extracted mRNA was then reverse transcribed to cDNA which was amplified using real time PCR. Briefly, 62.5 ng of cDNA was added to 0.4 µM pIgR primers (forward: CCACCGTGGAGATCAAGATT and reverse: CAGCCCGTGTTATTCCACTT primer) and QuantiTect SYBR Green PCR master mix to a final volume of 50 µl. The reaction mixture then went through 45 cycles in the Rotorgene 6000 Real time thermo cycler. Non-template controls were prepared routinely and analysed together with the samples. The reference gene used was CK19 (primer sequences: forward 5’-TCGACAACGCCCGTCTG; reverse 5’-CCACGCTCATGCGCAG).

**Effects of ATRA on α4β7 and CCR9 expression over time.**

Participants were randomized to receive Vivotif vaccine with or without 10 mg ATRA given orally an hour before vaccination and daily for 8 days. Blood was collected into EDTA and plain vacutainers (Becton Dickinson, Franklin Lakes, NJ, USA) at baseline, 6 hours, 24 hours, day 3, day 8 and day 14 post Vivotif vaccination and analyzed by flow cytometry.

Cells were stained for sorting as follows: A lyse-wash protocol (Becton Dickinson) was used to stain the blood samples. Briefly, 100μl of blood was stained with the different monoclonal antibodies. The following monoclonal antibodies were used for staining of whole blood: CD3-APC-H7 (SK7), CD4 PerCP (L200, α4-FITC (9F10), β7-APC (FIB504) and CCR9-PE (112509). Activated T cell subsets were then sorted using three different conjugated antibodies HLA-DR (G46-6), CD69 (FN50) and CD25 (M-A251). Tubes were vortexed and incubated at room temperature (25^o^C) in the dark (to improve fluorochrome activity) for 15 minutes. 2ml FACS lysing solution (Becton Dickinson) was added to the tubes after incubation, vortexed and incubated further for 10minutes. The tubes were then centrifuged at 264 x g for 5minutes and supernatant discarded leaving behind about 50ul. Cells were re-suspended by vortexing and washed in 2ml cell wash at 264 x g for 5minutes. Supernatant was discarded and tubes vortexed. Cells were fixed with 250ul cell fix (Becton Dickinson). Cells were counted on the FACSVerse machine using the FACSuite flow cytometry software (Becton Dickinson). Sequential gating to identify T cell subsets was used. Lymphocytes were identified and gated by their forward and side scatter (FSC and SSC). The CD3 positive T cells were then further identified and gated by the expression of CD4 and CD8. The relative expression of α4β7 and CCR9 to identify α4β7^+^ and CCR9^+^ gut homing cells were then identified on both the CD4 and CD8 populations

**Transcriptome analysis of effects of ATRA during vaccination**

Total RNA was extracted using the Paxgene blood RNA isolation kit (Qiagen), following standard kit protocol. Quantification and quality assessment of total RNA was performed using the 2100 Bioanalyzer (Agilent). cDNA libraries were prepared from total RNA using the Kapa Stranded RNA-seq Library Prep Kit (Roche). Libraries were sequenced on the HiSeq 2500 (Illumina), using Rapid v2 single read run of 100 bp-long sequence reads (+ adapter/index sequences). The FASTQ sequence reads were aligned to the hg19 human genome with TopHat2 version 2.1.1 [47] and mapped to Ensembl GRCh37 transcripts. Mapped reads were sorted and indexed using samtools 0.1.19 [48], followed by read-counts generation using htseq-count (HTSeq 0.6.0, [49]. All data processing and subsequent differential gene expression analyses were performed using R version 3.2.3 and DESeq2 version 1.10.1 [50]. Differential genes were identified using the likelihood-ratio test [50] and filtering for any genes that showed 1.5-fold change [log_2_(fold-change)] and adjusted p-value < 0.05 (cut-off at 5% false discovery rate (FDR)) as the threshold. Functional discovery of pathway enrichment and network analyses were performed using InnateDB and NetworkAnalyst respectively [28,29].
