# Supplementary material for: Retinoic acid elicits a coordinated expression of gut homing markers on T lymphocytes of Zambian men receiving oral Vivotif, but not Rotarix, Dukoral or OPVERO vaccines
Source: Vaccine. 2018 Jun 27;36(28):4134–41. doi: 10.1016/j.vaccine.2018.04.083 (PMC6020133; doi:10.1016/j.vaccine.2018.04.083)
Supplement: Supplementary data 2 [file mmc2.docx]

**SUPPLEMENTARY MATERIAL RESULTS**

**ATRA increased gut IgA directed at Vivotif LPS, but not other vaccine antigens**

There was no significant change in the levels of vaccine-specific serum IgA and IgG in any of the vaccine groups (Supplementary Figure 2). Together, these data suggested that the ATRA effect was mediated only in the mucosal compartment when given in conjunction with Vivotif vaccine and did not alter the responses to the other oral vaccines tested.

We assessed whether the increase in specific-IgA to Vivotif LPS was due to increased expression of the IgA transport molecule pIgR. Expression of the pIgR mRNA was assessed by RT-PCR. Our data revealed that there was no significant difference in expression of pIgR (pIgR:CK19 ratio, *P*>0.05) suggesting that the effect of ATRA was likely not due to changes in pIgR expression (Supplementary Figure 3).

**ATRA had different effects on gut homing marker expression on circulating CD4^+^T cells over time**

We next examined the gut homing receptor phenotype of circulating CD4^+^ T cells during oral vaccination in the presence or absence of ATRA. We quantified the number of activated CD4^+^ T cells (HLA-DR^+^ CD4^+^ T cells) that expressed integrin α4β7 and chemokine receptor CCR9 at six different time points after the administration of ATRA with or without Vivotif. In participants that received both Vivotif and ATRA, an increase of 68% in the total CD4^+^T cell count (P<0.001) and of 64% in CD4^+^T cells that expressed α4β7 (P=0.003) or 86% in cells that expressed CCR9 (P=0.0002) was observed at day 14 (Supplementary Figure 5). Counter-intuitively these increases did not precede the intestinal IgA response (Figure 1). Conversely, a slight decrease in α4β7^+^ DR^+^CD4 ^+^T cells was observed at day 3 (P=0.02) and day 8 (P=0.01) in participants that received both Vivotif and ATRA. Participants given either vaccine alone or ATRA alone showed a slight decrease in α4β7 expression (P=0.03 and P=0.01 respectively) at day14 (Supplementary Figure 5).

**Transcriptome analysis**

To elucidate the transcriptional changes that occurred during co-administration of ATRA and the oral vaccine Vivotif, we performed RNA-Seq on blood samples from eight male individuals, collected on day 0 (baseline, before vaccine and ATRA administration) and day 8 (after 3 doses of Vivotif and a course of ATRA treatment). Although substantial inter-individual variability was observed, 56 genes were commonly differentially expressed [Fold change (FC) of >1.5 or < -1.5 at an adjusted p-value of <0.05] when comparing the blood transcriptome on day 8 to that on day 0 (Supplementary Table 1).

We used pathway and network analysis tools to characterize the functional repertoire of these differentially expressed genes. We separately submitted up- and down-regulated genes to the innate immunity interactome database InnateDB [27], and analyzed pathways that are over-represented. Within the up-regulated genes, we observed enrichment of the complement cascade (adjusted *P*=0.001). In contrast, we observed enrichment in the interferon α/β signaling pathway (adjusted *P*=0.001), as well as membrane-ECM interactions (adjusted *P*=0.002) amongst the down-regulated genes. To gain systems level understanding of these differentially expressed genes, we performed network analysis using NetworkAnalyst [28,29]. NetworkAnalyst (http://www.networkanalyst.ca) uses the list of differentially regulated genes as seed proteins and mines the human protein-protein interaction (PPI) network. We identified several key immune hubs [hubs are highly interconnected nodes in networks that receive and distribute signals], including fibronectin 1 (FN1) whose attachment to B cells is directly affected by α4β7 [30], AXL (AXL Tyrosine-protein kinase receptor), complement component 1 (C1QB), complement component 4 binding protein (C4BPA), and HLA-DRB1 (Supplementary Figure 6). This network analysis suggests that the combined effect of ATRA and Vivotif has effects on B cell function (immunoglobulin genes), HLA-DR expression (increased), and IFN-α related genes (decreased).
