# Supplementary figures and images for: Retinoic acid elicits a coordinated expression of gut homing markers on T lymphocytes of Zambian men receiving oral Vivotif, but not Rotarix, Dukoral or OPVERO vaccines
Source: Vaccine. 2018 Jun 27;36(28):4134–41. doi: 10.1016/j.vaccine.2018.04.083 (PMC6020133; doi:10.1016/j.vaccine.2018.04.083)

## Slide 1
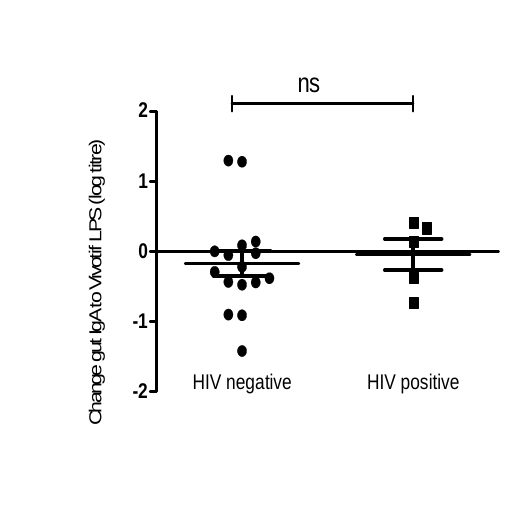

Supplement: Supplementary Fig. 1 — Changes in wglf IgA to Vivotif LPS in HIV seronegative and HIV seropositive vaccine recipients. No significant difference (P = 0.34) was seen in IgA responses between the two groups. [file mmc3.ppt]

## Slide 1
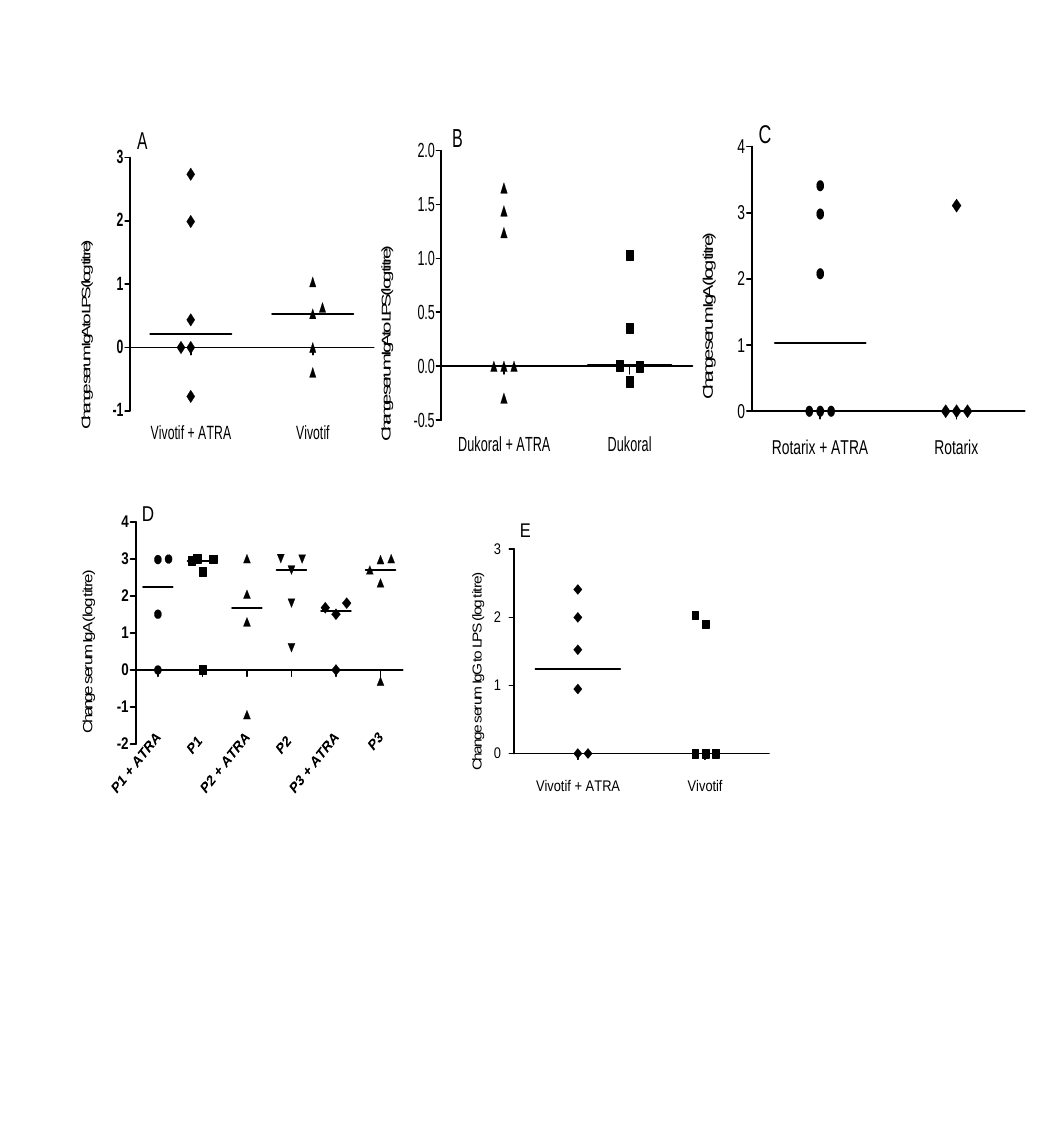

Supplement: Supplementary Fig. 2 — Change in specific IgA and IgG in serum of volunteers given one of 3 vaccines with or without ATRA. Antigen-specific IgA responses to Vivotif LPS, Dukoral LPS, Rotarix antigen and Opevero antigens were measured in serum. There was no significant difference in serum IgA in (A) Vivotif recipients who received vaccine with ATRA or no ATRA. No change was seen with the other vaccine antigens either (B) Dukoral LPS, (C) Rotarix antigen and (D) Opvero antigen. (E) Antigen-specific IgG responses in serum of Vivotif recipients showed no difference between the two groups. Opvero antigens: P1, P2 and P3. [file mmc4.ppt]

## Slide 1
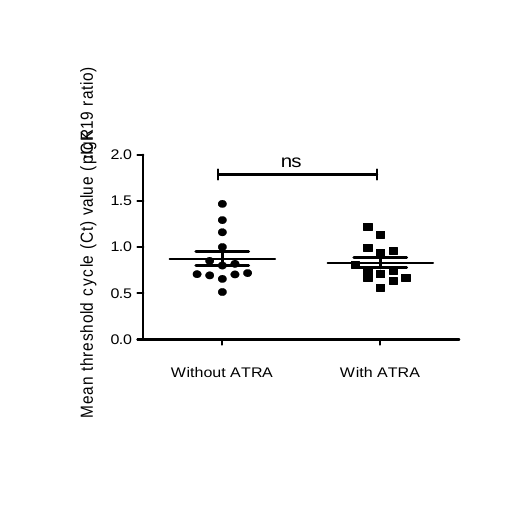

Supplement: Supplementary Fig. 3 — Summary of changes in pIgR:CK19 ratio. Effect of ATRA on pIgR was measured at baseline and day 14 post vaccination. There was no significant difference in the change in pIgR:CK19 ratio post Vivotif vaccination alongside (or without) ATRA treatment. [file mmc5.ppt]

## Slide 1
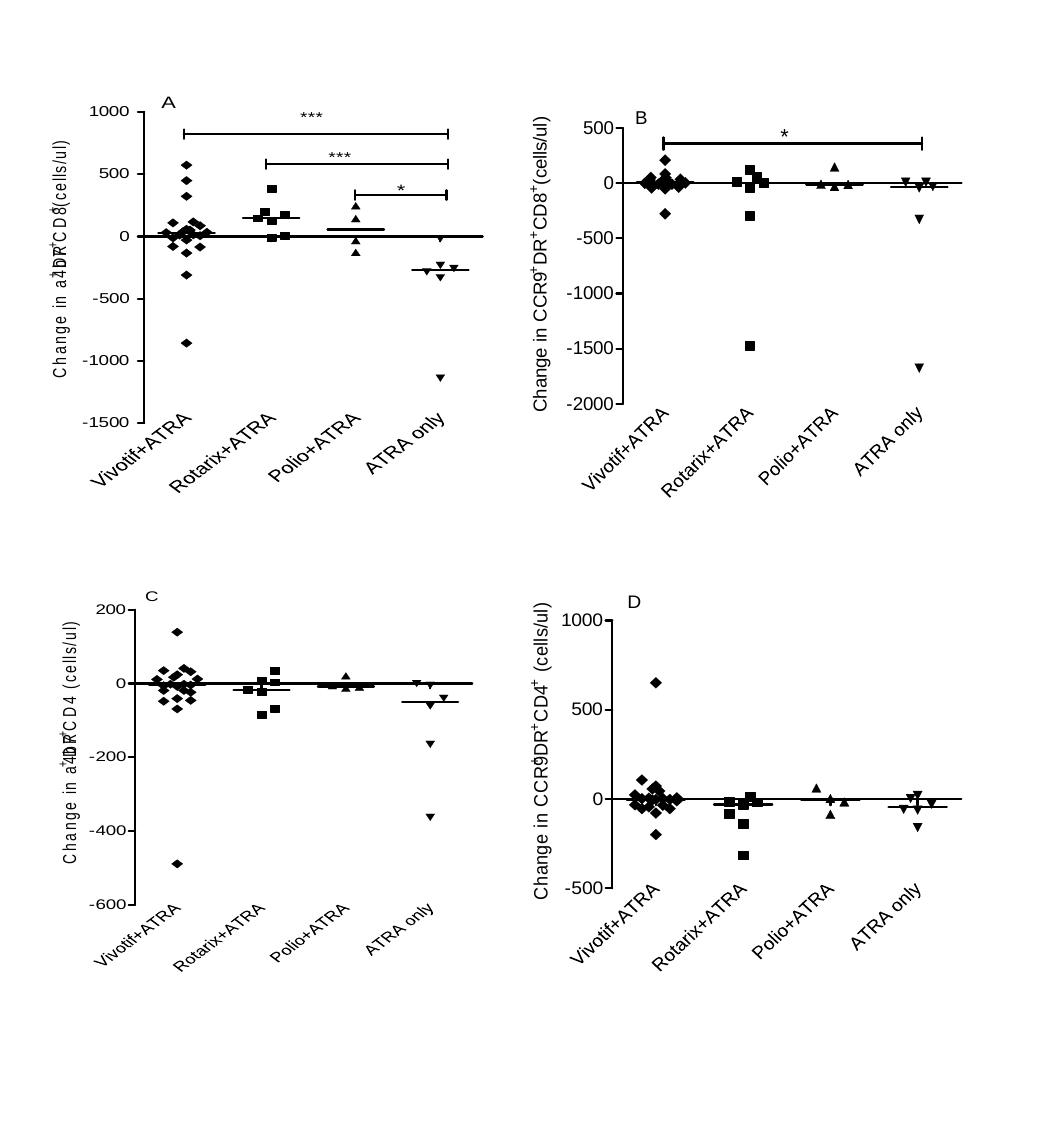

Supplement: Supplementary Fig. 4 — Summary of changes on activated CD4+ and CD8+T cells expressing α4β7 and CCR9 in volunteers given one of 3 vaccines (Vivotif, Rotarix or Opvero) plus ATRA and those given ATRA alone.(A)Change in α4β7 expression on DR+CD8+ T cells was significantly higher in participants that received vaccine with ATRA: Vivotif with ATRA (∗∗∗P = 0.004), Rotarix with ATRA (∗∗∗P = 0.001) and polio with ATRA (∗P = 0.04).(B) Change in CCR9 expression on DR+CD8+ T cells was significantly higher only in the those that received Vivotif with ATRA (∗P = 0.03).There was no difference in (C) α4β7 or (D) CCR9 expression on DR+CD4+ T cells for all of the vaccine group. [file mmc6.ppt]

## Slide 1
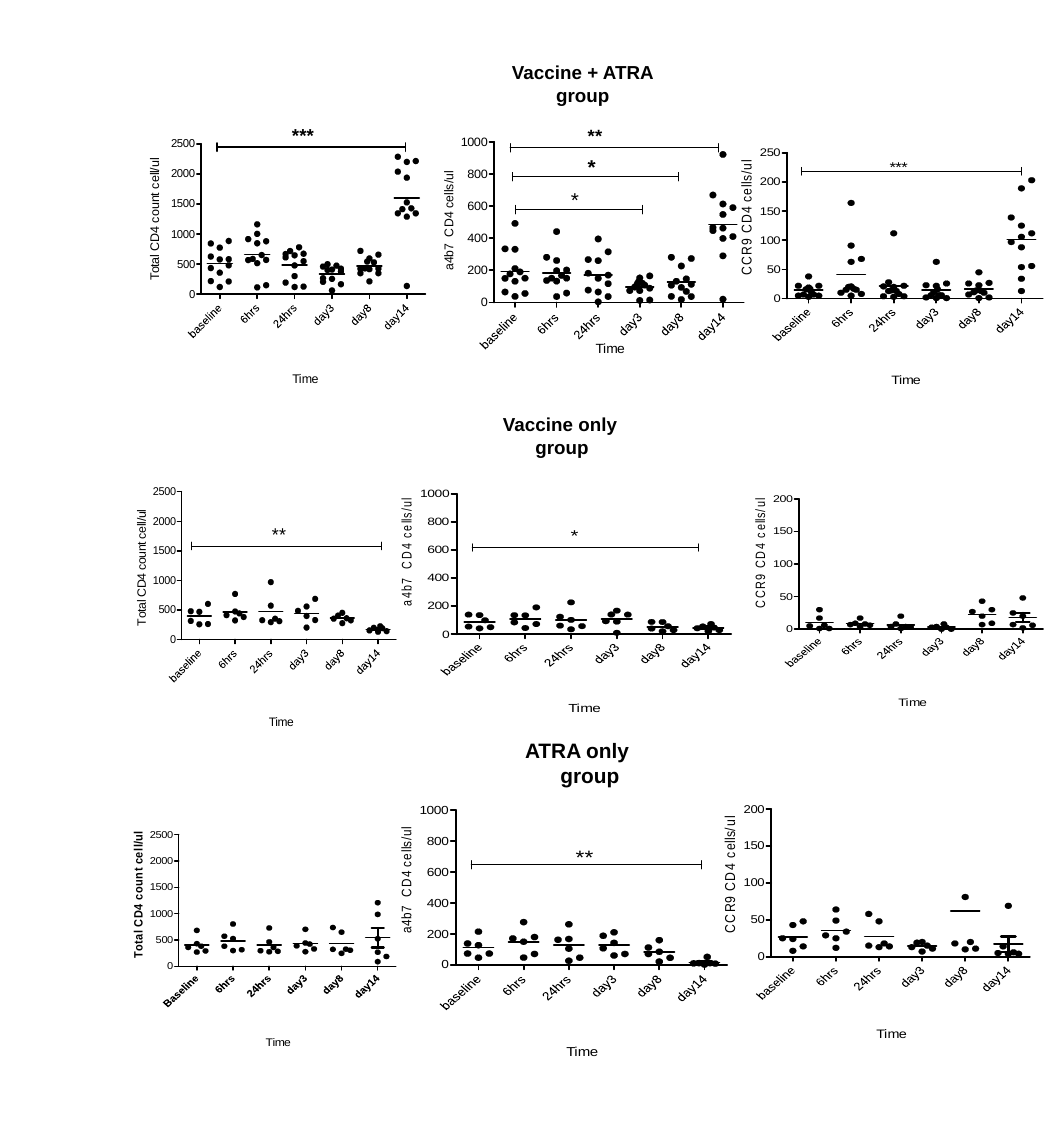

Vaccine + ATRA
group
Vaccine only
group
ATRA only
group

Supplement: Supplementary Fig. 5 — 10 mg ATRA given daily for 8 days following oral Vivotif vaccination enhanced total CD4+ T cell count, α4β7-integrin and CCR9 on gut homing lymphocytes. Participants given vaccine with ATRA showed a drop in α4β7 expression at day 3 (∗P = 0.021) and day 8 (∗P = 0.013) but increased at day 14 (∗P = 0.003). CCR9 expression (∗∗∗P = 0.0002) and total CD4 count (∗∗P < 0.0001) was also increased in the same group. Participants given vaccine only and ATRA only showed a drop in α4β7 expression (∗P = 0.025 and ∗∗P = 0.009 respectively) over 14 days. The total CD4 count in the vaccine only group also showed a significant drop (∗∗P = 0.001) at day 14. Asterisks (∗) indicate that the effect of ATRA over the indicated time course is statistically significant . Effects of ATRA over time were analsed using the 1-way ANOVA test. [file mmc7.ppt]

## Slide 1
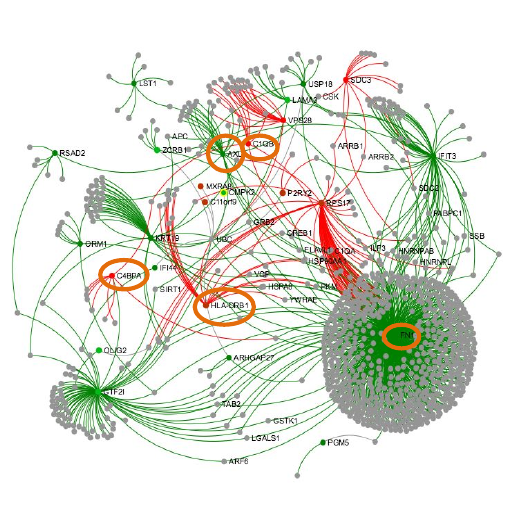

Supplement: Supplementary Fig. 6 — Network analysis of differentially expressed genes using NetworkAnalyst. 56 differentially expressed genes identified in the blood of volunteers, by RNA-Seq, were analyzed by comparing day8 versus baseline (pre-immunization) of ATRA treatment alongside vaccination (identified by DESeq2 likelihood ratio test). Red genes are upregulated and green genes are downregulated and grey nodes are direct interactors of these genes. Key immune hubs identified include fibronectin 1 (FN1), AXL Tyrosine-protein kinase receptor, complement component 1(C1QB), complement component 4 binding protein (C4BPA) and HLA-DRB1 are highlighted with an orange ring. [file mmc8.ppt]
