# Supplementary material for: Retinoic acid elicits a coordinated expression of gut homing markers on T lymphocytes of Zambian men receiving oral Vivotif, but not Rotarix, Dukoral or OPVERO vaccines
Source: Vaccine. 2018 Jun 27;36(28):4134–41. doi: 10.1016/j.vaccine.2018.04.083 (PMC6020133; doi:10.1016/j.vaccine.2018.04.083)
Supplement: Supplementary Table 1 [file mmc9.docx]

**Supplementary Table 1. Differentially expressed genes during vaccination and ATRA treatment**

| **Ensembl ID** | **Gene Name** | **Pvalue (adj)** | **FoldChange** | **Function/description** |
| --- | --- | --- | --- | --- |
| ENSG00000262087 | LILRB3 | 9.06E-26 | 1.64 | Leukocyte Ig-like receptor B3 |
| ENSG00000239998 | LILRA2 | 0.020 | 1.56 | Leukocyte immunoglobulin-like receptor 7 |
| ENSG00000262320 | LILRA6 | 0.00039 | 1.53 | Leukocyte immunoglobulin-like receptor A6 |
| ENSG00000211965 | IGHV3-49 | 0.0068 | -2.80 | IgHvariable region 3-49 |
| ENSG00000270597 | IGHV3-48 | 0.00037 | -1.82 | IgHvariable region 3-48 |
| ENSG00000211934 | IGHV1-2 | 0.041 | 1.60 | IgHvariable region 1-2 |
| ENSG00000231475 | IGHV4-31 | 0.023 | 1.72 | IgHvariable region 4-31 |
| ENSG00000225824 | HLADQB | 1.88E-12 | 1.64 | Major histocompatibility complex (MHC), class II antigen DQ beta |
| ENSG00000229074 | HLADRB1 | 9.02E-11 | 1.66 | MHC Class II HLA-DR-Beta Cell Surface Glycoprotein |
| ENSG00000231939 | HLADQB1 | 1.62E-09 | 1.65 | Major histocompatibility complex (MHC), class II antigen DQ beta-1 |
| ENSG00000119917 | IFIT3 | 2.13E-06 | -1.62 | Interferon induced;RIG-G; Antiviral; Increased by ATRA in APL |
| ENSG00000137959 | IFI44L | 4.80E-06 | -1.53 | Interferon induced |
| ENSG00000137965 | IFI44 | 0.00010 | -1.69 | Interferon induced |
| ENSG00000134326 | CMPK2 | 4.08E-09 | -1.66 | Cytidine monophosphate kinase 2 |
| ENSG00000206433 | LST1 | 0.00093 | -1.84 | Leucocyte-specific transcript 1 |
| ENSG00000197582 | GPX1P1 | 0.0016 | -2.31 | Glutathione peroxidase 1 pseudogene 1 |
| ENSG00000202198 | RN7SK | 0.0022 | 2.05 | snRNA |
| ENSG00000175591 | P2RY2 | 0.0034 | 1.52 | Purinergic receptor |
| ENSG00000154330 | PGM5 | 0.0056 | -1.51 | Phosphoglucomutase 5 |
| ENSG00000161055 | SCGB3A1 | 0.0067 | -1.86 | Secretoglobin |
| ENSG00000235373 | lncRNA | 0.0085 | 1.62 | Long non-coding RNA |
| ENSG00000248810 | lncRNA | 0.0093 | -1.63 | Long non-coding RNA |
| ENSG00000167601 | AXL | 0.0094 | -2.43 | Receptor tyrosine kinase |
| ENSG00000238268 | RP11-229P13.19 | 0.0095 | 1.68 | Long intergenic non-coding RNA (lincRNA) |
| ENSG00000205246 | RPSAP58 | 0.012 | -1.83 | Also acts as laminin receptor |
| ENSG00000204677 | FAM153C | 0.013 | 1.97 | Pseudogene |
| ENSG00000168913 | ENHO | 0.013 | 1.61 | Energy homeostasis associated |
| ENSG00000177359 | OVOS2 | 0.015 | 1.50 | Ovostatin2 |
| ENSG00000171766 | GATM | 0.016 | 1.59 | Glycine amidinotransferase |
| ENSG00000139168 | ZCRB1 | 0.021 | -1.55 | Zinc finger CCHC-type and RNA binding motif containing 1 |
| ENSG00000273112 | RP11-25K21.6 | 0.029 | -1.63 | lncRNA |
| ENSG00000257961 | AC003070.1 | 0.033 | -1.58 | Rho GTPase-activating protein 27 isoform a |
| ENSG00000197620 | CXorf40A | 0.039 | 1.60 | Chromosome X open reading frame 40A |
| ENSG00000162576 | MXRA8 | 0.041 | 1.54 | Matrix-remodelling associated 8 |
| ENSG00000250896 | RNPS1P1 | 0.042 | 4.29 | RNA binding protein S1, Pseudogene |
| ENSG00000162512 | SDC3 | 0.044 | 1.52 | Syndecan 3 |
| ENSG00000205147 | AC016586.1 | 0.046 | -1.68 | Long intergenic non-coding RNA (lincRNA) |
| ENSG00000173369 | C1QB | 0.027 | 1.53 | Complement component 1, q subunit, B chain |
| ENSG00000123838 | C4BPA | 2.13E-10 | 1.66 | Complement 4 binding protein |
| ENSG00000215764 | KIR3DS1 | 3.23E-37 | 1.51 | Killer cell Ig-like receptor |
| ENSG00000184979 | USP18 | 5.38E-05 | -2.24 | Ubiquitin specific peptidase 18 |
| ENSG00000134321 | RSAD2 | 8.78E-06 | -2.27 | Interferon inducible antiviral |
| ENSG00000260313 | VPS28 | 0.0025473 | 1.68 | Vesicular protein sorting; Antiviral; Viral Ag processing |
| ENSG00000167236 | CCL23 | 6.27E-05 | 1.58 | Chemokine |
| ENSG00000233426 | EIF3FP3 | 0.00014 | 2.21 | Eukaryotic translation initiation factor 3, subunit F pseudogene 3 |
| ENSG00000184779 | RPS17 | 0.0041 | 1.52 | Ribosomal protein |
| ENSG00000233913 | RPL10P9 | 0.00034 | 1.75 | Ribosomal protein pseudogene |
| ENSG00000263001 | GTF2I | 0.00032 | -1.81 | Transcription factor 2i; increases PI3K, TGFβ, serotonin receptor3a |
| ENSG00000115414 | FN1 | 0.0020 | -1.70 | Fibronectin precursor |
| ENSG00000053747 | LAMA3 | 0.044 | -2.53 | Laminin subunit alpha 3 |
| ENSG00000115155 | OTOF | 1.52E-16 | -2.88 | Otoferlin; putative function in vesicle fusion |
| ENSG00000124920 | MYRF | 4.96E-10 | 1.72 | Myelin regulatory factor; required for oligodendrocytes |
| ENSG00000205927 | OLIGO2 | 0.00012 | -1.60 | Oligodendrocyte transcription factor 2 |
| ENSG00000229314 | ORM1 | 0.011 | -1.57 | Orosomucoid |
| ENSG00000171345 | KRT19 | 0.0034 | -1.77 | Cytokeratin 19 |
| ENSG00000088827 | SIGLEC1 | 2.12E-06 | -1.67 | Sialo-adhesin; macrophage binding to lymphocytes |
